# Implication of Non-electrostatic Contribution to Deionization in Flow-Electrode CDI: Case Study of Nitrate Removal From Contaminated Source Waters

**DOI:** 10.3389/fchem.2019.00146

**Published:** 2019-03-22

**Authors:** Jingke Song, Jinxing Ma, Changyong Zhang, Calvin He, T. David Waite

**Affiliations:** ^1^UNSW Water Research Centre, School of Civil and Environmental Engineering, University of New South Wales, Sydney, NSW, Australia; ^2^College of Environmental Science and Engineering, Tongji University, Shanghai, China; ^3^Shanghai Institute of Pollution Control and Ecological Security, Shanghai, China

**Keywords:** flow-electrode, capacitive deionization, nitrate removal, energy consumption, source waters, water recovery

## Abstract

While flow-electrode capacitive deionization (FCDI) operated in short-circuited closed cycle (SCC) mode appears to hold promise for removal of salt from brackish source waters, there has been limited investigation on the removal of other water constituents such as nitrate, fluoride or bromide in combination with salt removal. Of particular concern is the effectiveness of FCDI when ions, such as nitrate, are recognized to non-electrostatically adsorb strongly to activated carbon particles thereby potentially rendering it difficult to regenerate these particles. In this study, SCC FCDI was used to desalt source waters containing nitrate at different concentrations. Results indicate that nitrate can be removed from source waters using FCDI to concentrations <1 mg NO_3_-N L^−1^ though a lower quality target such as 10 mg L^−1^ would be more cost-effective, particularly where the influent nitrate concentration is high (50 mg NO_3_-N L^−1^). Although studies of the fate of nitrate in the FCDI system show that physico-chemical adsorption of nitrate to the carbon initially plays a vital role in nitrate removal, the ongoing process of nitrate removal is not significantly affected by this phenomenon with this lack of effect most likely due to the continued formation of electrical double layers enabling capacitive nitrate removal. In contrast to conventional CDI systems, constant voltage mode is shown to be more favorable in maintaining stable effluent quality in SCC FCDI because the decrease in electrical potential that occurs in constant current operation leads to a reduction in the extent of salt removal from the brackish source waters. Through periodic replacement of the electrolyte at a water recovery of 91.4%, we show that the FCDI system can achieve a continuous desalting performance with the effluent NO_3_-N concentration below 1 mg NO_3_-N L^−1^ at low energy consumption (~0.5 kWh m^−3^) but high productivity.

## Introduction

In recent decades, nitrate has become one of the most serious problems affecting water quality globally as a result of the intensification of agricultural activities, particularly with regard to the use of fertilizers (Lado et al., 2017; Oyarzun et al., 2018). While nitrate itself is not particularly toxic, critical concerns exist in the reduction of nitrate in the human digestive system to toxic nitrite (Ward, 2005). Several studies have indicated that the occurrence of methaemoglobinaemia to which infants are especially susceptible has a direct relationship with elevated levels of nitrate in drinking water (Sadler et al., 2016). As a result, the maximum contaminant level (MCL) for NO_3_-N in drinking water has been set at 10 mg L^−1^ by the U.S. Environmental Protection Agency and 50 mg L^−1^ as nitrate (equivalent to 11 mg L^−1^ as NO_3_-N) by the World Health Organization (Ward, 2005; Wang and Chu, 2016). In addition, the presence of nitrate in water bodies can stimulate the excessive growth of algae and other aquatic plants resulting in harm to river and lake ecosystems. In New Zealand, an updated freshwater guideline suggests that 1.0 mg NO_3_-N L^−1^ is the limiting chronic nitrate exposure value for pristine environments with high biodiversity (Hickey, 2013).

Of the nitrate removal technologies available, biological denitrification is an environmentally friendly and cost-effective method and has been widely applied in biofilters to polish source waters but its efficiency can be significantly affected by a variety of parameters including water temperature, alkalinity and pH. In most cases, the effluent quality from such a process, particularly when operated at high denitrification rates, does not meet the stricter standards (e.g., 1.0 mg NO_3_-N L^−1^) now being either imposed or suggested (Ghafari et al., 2008; Karanasios et al., 2010). Alternatively, physico-chemical technologies such as reverse osmosis (RO) (Schoeman and Steyn, 2003), electrodialysis (ED) (Kikhavani et al., 2014), and ion exchange (Ma et al., 2012) can be employed for physico-chemical removal of nitrate from source waters. While high removal rates have been achieved by these methods, the requirement for relatively extreme conditions (e.g., high pressure for RO and high voltage for ED) increases the operating cost. Moreover, the ion exchange method may generate secondary pollution during the regeneration of used sorbents (Kim and Choi, 2012).

In recent years, there has been considerable progress in the electrical extraction of ions from brackish waters at low voltages (<1.2~1.6 V) followed by ion immobilization in electrical double layers (EDLs) on carbon electrodes, with one of the recent innovations, flow electrode capacitive deionization (FCDI), gaining in popularity (Jeon et al., 2013; Hatzell et al., 2015; Rommerskirchen et al., 2015; Ma et al., 2016; Yang et al., 2016). Compared to capacitive deionization (CDI) using a solid electrode, continuous desalting can be achieved in FCDI, largely because of the infinite ion-adsorption capacity that can be achieved by pumping uncharged carbon materials into the system (Suss et al., 2015; Doornbusch et al., 2016; Yang et al., 2017). In practical applications, a short-circuited method can be implemented to regenerate the flow electrode in a closed cycle with this process resulting in minimization of the amount of carbon materials used (Yang et al., 2017). Another advantage of the electrosorption process relates to the negligible chemical consumption required to maintain the adsorption capacity for charged species in long-term operation (Seo et al., 2010; Zhang et al., 2018a,b). Recent studies have shown the applicability of CDI for selective nitrate removal from dilute streams with the efficiency further improved with the use of composite electrode materials (Kim and Choi, 2012; Tang et al., 2015; Oyarzun et al., 2018). In contrast, there has been, surprisingly, no investigation of the removal of nitrate using FCDI despite the fact that, given the continuous migration of nitrate and other ions into the flow electrode, high nitrate removal efficiency and water recovery could well be achieved.

In a typical (F)CDI system, electrosorption of ions into EDLs is considered the primary means of salt removal from the brackish stream with the continuous operation of FCDI in short-circuited closed cycle (SCC) mode relying on the full discharge of the flow electrode in the shared recirculation reservoir (Yang et al., 2017). However, carbon is a well-known sorbent, with adsorption occurring non-electrostatically by physico-chemical mechanisms. A number of studies have shown that the physico-chemical removal of nitrate can be induced by the addition of activated carbon into aqueous solutions (OZtürk and Bektaş, 2004; Demiral and Gündüzoglu, 2010). As such, it is possible that, following migration across the membrane, nitrate is preferentially adsorbed to the carbon particles, resulting in difficulty in short-circuited operation in fully recovering the adsorption capacity of the flow electrode. Of particular concern is the possibility that deterioration in efficiency of removal of nitrate might occur over time. As such, a comprehensive understanding of the fate of nitrate in FCDI is of considerable importance for better implementation of this technology in potable water production.

In this study, different concentrations of NO_3_-N (50, 20, and 10 mg L^−1^) were used to simulate source waters that have been contaminated to different levels (Lasagna et al., 2016). An FCDI system was operated in single-pass mode with the flow electrodes recirculated in short-circuited closed cycle. The impacts of current density and hydraulic retention time (HRT) on the process performance were investigated with consideration given to the mechanisms of nitrate removal. Based on the resulting conclusions, viable strategies have been proposed for the continuous operation of FCDI.

## Materials and Methods

### Materials and Reagents

All chemicals were purchased from Sigma-Aldrich (Castle Hill NSW, Australia) unless otherwise mentioned. Activated charcoal (Darco, 100 mesh, with an average pore size and BET surface area of 1.44 nm and 917.5 m^2^ g^−1^, respectively) blended with carbon black at a ratio of 9:1 was used as the carbon material. Each flow electrode contained 8 g of carbon particles in a 1,000 mg L^−1^ NaCl solution (80 g in total) with this carbon load representing 10 *wt*%. Note that carbon black (500 mS cm^−1^, 1 bar) was used to facilitate the charge transfer within the carbon materials though the ohmic resistance of the flow electrodes is largely dominated by the ionic resistance of the electrolyte. Simulated source water was prepared by mixing an appropriate amount of NaNO_3_ and NaCl in Milli-Q water to achieve an initial NO_3_-N concentration of 50, 20, or 10 mg L^−1^ with a fixed NaCl concentration of 1,000 mg L^−1^.

### Experimental Setup

**Figure 1:**
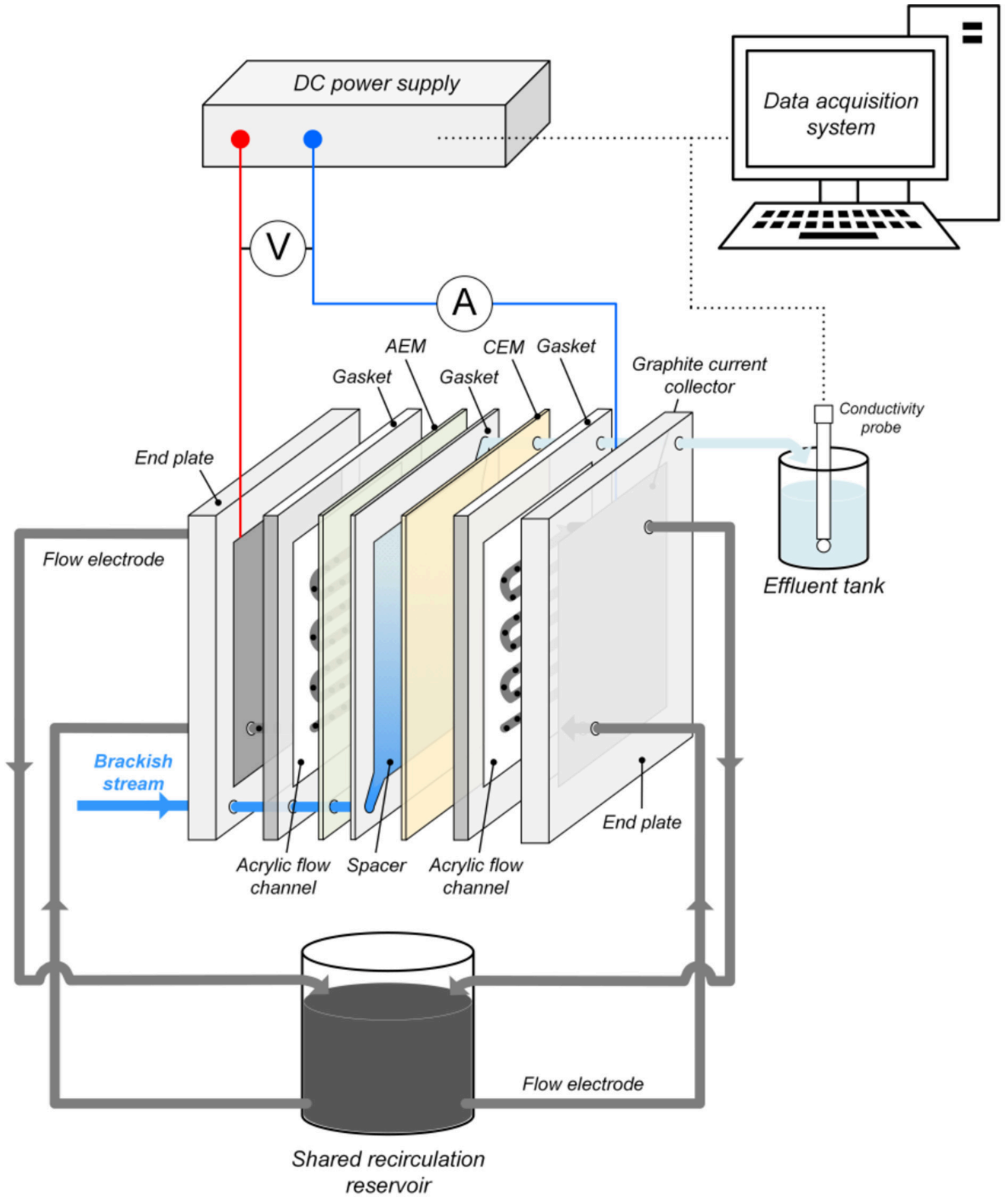
Schematic representation of the FCDI system for nitrate removal with anion and cation exchange membranes (AEM and CEM, respectively) separating the feed stream from the flow electrode suspensions.

Figure 1 shows the structure of the FCDI cell used in all experiments. The composite flow chambers for the slurry electrode consisted of graphite current collectors and carved serpentine acrylic flow channels (3 mm wide and 3 mm deep resulting in an effective contact area *A*_*eff*_ = 34.9 cm^2^ between the flow electrode and ion exchange membrane from the inlet to the outlet). Ion exchange membranes (CEM-Type I/AEM-Type I, FUJIFILM Europe) were placed, respectively, against the channels to separate the flow chambers and the spacer (Figure 1). Simulated water passed through a 0.5 mm thick spacer (100 mesh) made of nylon sheet located between the ion exchange membranes, with silicone gaskets encircling the spacer channel in order to avoid the leakage of the solution. All parts of the FCDI cell were held together using acrylic end plates (Figure 1).

### FCDI Operation and Sample Measurement

In all FCDI experiments, the flow electrode was recirculated in SCC mode using a peristaltic pump (Longer pump, Baoding, China) with the flow rate fixed at 80 mL min^−1^ (i.e., 40 mL min^−1^ each) (Figure 1). Constant current/voltage was provided by a DC power supply (MP3840, Powertech). The current density was calculated from *i* = *I*/*A*_*eff*_ where *I* is the current (A) and *A*_*eff*_ is the effective area between the flow electrode and ion exchange membrane (34.9 cm^2^). The electrical conductivity of the effluent was measured using a conductivity meter (CON-BTA, Vernier) connected to a data acquisition system (SensorDAQ, Vernier). The concentrations of nitrate-N (NO_3_-N) in aqueous solutions were determined using a UV screening method published by the American Public Health Association (Rice et al., 2012). Prior to measurement of aqueous nitrate concentrations in the flow electrode samples, 0.22 μm syringe filters were used to separate the solution and activated carbon particles.

#### Ion Removal in Different Scenarios

The FCDI performance with regard to nitrate and salt removal from solutions containing 50, 20, and 10 mg NO_3_-N L^−1^ (and 1,000 mg NaCl L^−1^) were initially evaluated at various constant current densities (*i* = 1.4~30.1 A m^−2^). Because the positively (termed anode) and negatively (termed cathode) charged flow electrodes were recirculated to the shared reservoir (Figure 1) for charge neutralization and electrode re-generation after passing through the flow chambers, no extra discharging step was used in this work. Due to the short operation time (20~30 min) of FCDI, the effect of ion accumulation in the flow electrodes on desalting performance was not considered. The brackish water was fed into the spacer chamber in single pass mode with the flow rate (*q*_0_) changing from 0.85 to 3.40 mL min^−1^, corresponding to a hydraulic retention time (HRT) from 2.94 to 0.73 min. When the conductivity of the effluent became stable (typically, 10~20 min after setup), 5 mL samples were withdrawn from the effluent and analyzed to quantify the concentrations of NO_3_-N. The energy consumption as a function of productivity (kWh m^−3^) was obtained by integrating the cell voltage and current over the flow rate, as described by Equation (1):

(1) $$\begin{array}{r} E\;=\;\frac{IU_{cell}}{q_{0}} \end{array}$$

#### Non-electrostatic Adsorption of Nitrate Onto the Membrane and Carbon

The impact of non-electrostatic adsorption of nitrate in FCDI was investigated by changing the solution in the electrode channels (and the recirculation reservoir). In these experiments, the flow rate of the brackish stream containing 10 mg NO_3_-N L^−1^ and 1,000 mg NaCl L^−1^ was set as 2.55 mL min^−1^ (i.e., HRT = 0.98 min). The FCDI system firstly ran for 3,600 s without use of the flow electrodes to characterize the adsorption of nitrate onto the ion-exchange membrane. Subsequently, a 72 mL “control” flow electrode containing 1,000 mg NaCl L^−1^ was fed into the system in order to evaluate the diffusion of nitrate into the liquid phase in the absence of an electrical field. Assuming that the physico-chemical adsorption capacity of the membrane for NO_3_-N is fixed (*Q*_*e, mem*_, mg NO_3_-N m^−2^), the adsorption process may be considered to be pseudo-first order (Ho and Mckay, 1998) as described in Equations (2) and (3):

(2) $$\begin{array}{rcl} \;\frac{\text{d}Q_{t,mem}}{\text{d}t} & = & \;k_{1}\left(Q_{e,mem}-Q_{t,mem}\right) \end{array}$$

(3) $$\begin{array}{rcl} Q_{t,mem} & = & \;\frac{\int q_{0}\left(C_{0}\;-\;C_{t}\right)\text{d}t}{A_{mem}}\;\approx \;\frac{\sum_{j=1}^{t}\left(C_{0}\;-\;\frac{C_{j}\;+\;C_{j-1}}{\text{2}}\right)q_{0}t_{j}}{A_{mem}} \end{array}$$

where *Q*_*t, mem*_ (mg NO_3_-N m^−2^) is the dynamic adsorption capacity at time *t, k*_1_ is the pseudo-first-order rate constant (s^−1^), *q*_0_ = 2.55 mL min^−1^, *C*_0_ and *C*_*t*_ (mg L^−1^) indicate the initial NO_3_-N concentration (10 mg L^−1^) and concentration at time *t, j* presents the sampling point over the experiment and *A*_*mem*_ is the total surface area of the ion exchange membrane (112 cm^2^). Integrating Equation (2) for the boundary conditions (*t* = 0 to *t* = *t* and *Q*_*t, mem*_ = 0 to *Q*_*t, mem*_ = *Q*_*e, mem*_) yields (Equation 4):

(4) $$\begin{array}{r} Q_{t,mem}=\;Q_{e,mem}\left(\text{1}-e^{-k_{\text{1}}t}\right) \end{array}$$

With regard to the non-electrostatic uptake of NO_3_-N by the carbon particles in the flow electrode, the theoretical increase in the nitrate-N concentration in the flow electrode over time *t* (*X*_*tot*_, mg L^−1^) was calculated according to the mass balance (Equation 5). As such, the contribution of membrane adsorption to NO_3_-N removal at time *t* (*X*_IEM_, mg L^−1^) was normalized by the liquid volume (*V*_*flow*_ = 72 mL) of the flow electrode as follows Equation (6):

(5) $$\begin{array}{r} X_{tot}=\frac{\int q_{0}\left(C_{0}\;-\;C_{t}\right)dt}{V_{flow}} \end{array}$$

(6) $$\begin{array}{r} X_{\text{IEM}}=\frac{Q_{e,mem}\left(1-e^{-k_{1}t}\right)A_{mem}}{V_{flow}} \end{array}$$

The physico-chemical adsorption of nitrate in the flow electrodes was *ex-situ* evaluated at room temperature. Certain amounts of carbon electrodes were dispersed into 10 mL solutions containing 1,000 mg NaCl L^−1^ and various concentrations of NO_3_-N. Non-electrostatic adsorption (*Q*_*e, carbon*_, mg NO_3_-N g^−1^) of nitrate onto the carbon electrode particles was analyzed by fitting the results to a Langmuir isotherm model as follows (Demiral and Gündüzoglu, 2010) (Equation 7):

(7) $$\begin{array}{r} Q_{e,carbon}=\frac{Q_{\infty }bC_{e}}{1\;+\;bC_{e}} \end{array}$$

where *Q*_∞_ is the maximum adsorption capacity estimated by the Langmuir model (mg NO_3_-N g^−1^), *C*_*e*_ is the equilibrium concentration of NO_3_-N (mg L^−1^) and *b* is the Langmuir adsorption equilibrium constant (L mg^−1^). The non-electrostatic contribution of the flow electrodes to nitrate removal (*X*_*carbon*_) can be determined with the incorporation of Equation (8):

(8) $$\begin{array}{r} X_{carbon}=\frac{Q_{e,carbon}m}{V_{flow}}=\frac{\frac{Q_{\infty }bX_{free}}{1\;+\;bX_{free}}m}{V_{flow}} \end{array}$$

where *m* is the mass of the active materials (i.e., 7.2 g) in the flow electrode and *X*_*free*_ is the free nitrate-N concentration in the recirculation reservoir following electrode discharging. Since *X*_*free*_ relates to the nitrate removed due to the capacitive (and electrodialytic) mechanism, the synthesis of *X*_IEM_, *X*_*carbon*_, and *X*_*free*_ should account for *X*_*tot*_.

#### Critical Parameters Influencing Continuous Performance of the FCDI System

Comparison of the SCC FCDI performance in the constant current (CC) mode and constant voltage (CV) mode was conducted, particularly with regard to the fate of nitrate (and other ions) after migration across the membrane. A constant current *i* = 18.6 A m^−2^ (or voltage *U*_*cell*_ = 1.0 V) was applied during electrosorption with the nitrate concentrations in the effluent and flow electrode measured at predetermined intervals. To avoid accumulation of ions in the liquid phase of the flow electrode leading to ion back diffusion and leakage, aliquots of the electrolyte in the flow electrode (Δ*V*_*ele*_) were replaced by 1,000 mg L^−1^ NaCl solutions after every 5-h of electrosorption. The water recovery rate (γ) for FCDI is defined as follows Equations (9) and (10):

(9) $$\begin{array}{r} \gamma \;=\frac{\int qdt}{\int qdt\;+\;\Delta V_{ele}} \end{array}$$

(10) $$\begin{array}{r} \int qdt\;\approx \;q_{0}\int \text{d}t-\left(V_{ele,t}-V_{ele,0}\right) \end{array}$$

where *q* is the flow rate of the desalted water at time *t*. Because of the constant water transfer from the spacer into the flow chambers in SCC operation of FCDI (Yang et al., 2017), *q* should be slightly lower than the influent flow rate *q*_0_. Therefore, the desalted water yield was estimated according to Equation (10) where *V*_*ele*__, 0_ and *V*_*ele*__, t_ are the volumes of the electrolyte in the flow electrode (72 mL in this study) initially and at time *t*, respectively.

## Results and Discussion

### Salt and Nitrate Removal for Different Scenarios

**Figure 2:**
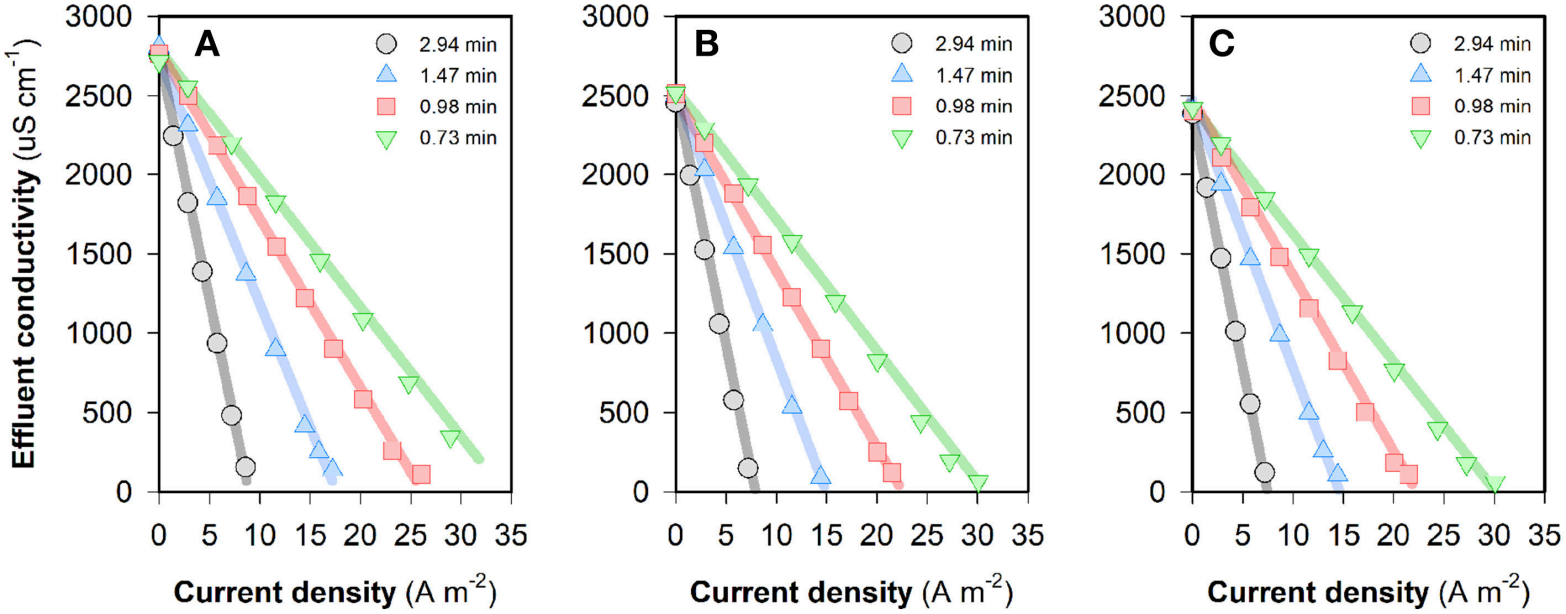
Change of the effluent conductivity by using FCDI when the feed water contains **(A)** 50, **(B)** 20, or **(C)** 10 mg NO_3_-N L^−1^. All experiments were conducted in a single-pass, constant-current mode. Flow electrode = 10 *wt*% carbon in 1,000 mg NaCl L^−1^ electrolyte. The legends in the figures indicate different HRTs. The operating time for electrosorption is 1,200~1,800 s. Lines serve to guide the eye.

The desalting performance of the FCDI system fed with source waters of different composition is shown in Figure 2. For a certain type of water treated at a fixed HRT, the “steady-state” effluent conductivity decreases with increase in the current density. It can be seen from Figure 2 that the influent nitrate concentration does not affect the desalination rate significantly. While higher ion removal rates can be obtained at longer HRTs, the water productivity is compromised. According to World Health Organization guidelines, water containing TDS concentrations below 1,000 mg L^−1^ (~2,000 μS cm^−1^) is usually acceptable to consumers, although the palatability of water has been rated by panels of tasters as excellent if the TDS is <300 mg/L and good if the TDS is between 300 and 600 mg/L (World Health Organization, 1996). Results in Figure 2 show that the FCDI system is capable of reducing the TDS concentration to a very low level, though very low concentrations in the spacer (i.e., conductivity <100 μS cm^−1^) should be avoided as this would lead to a dramatic increase in operating voltage and energy consumption (Figure S1). For example, when an influent NO_3_-N concentration of 10 mg L^−1^ and HRT of 0.98 min were used, the effluent conductivity of the system decreased from 125.1 to 50.3 μS cm^−1^ with the current density increasing from 20.1 to 21.5 A m^−2^ (Figure 2). However, the cell voltage increased sharply from 1.00 to 1.88 V (Figure S1). The relationship between the energy consumption and “steady-state” effluent conductivity is shown in Figure S2. Overall, it can be concluded that an increase in either the treatment efficiency (i.e., lower effluent conductivity) or the productivity (i.e., shorter HRT) would lead to an increase in energy consumption.

**Figure 3:**
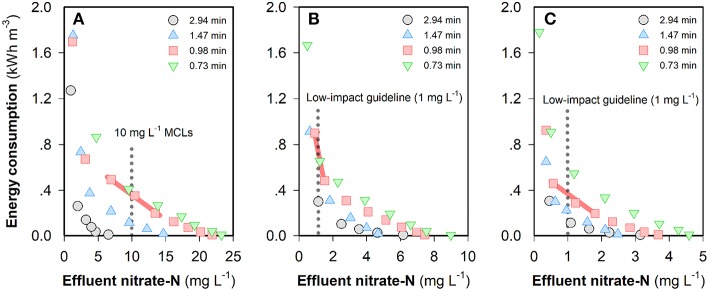
Energy consumption to obtain different “steady-state” NO_3_-N concentrations when the feed water contains **(A)** 50, **(B)** 20, or **(C)** 10 mg NO_3_-N L^−1^. The legends in the figures indicate different HRTs. Experimental conditions: single-pass, constant-current mode, flow electrode = 10 *wt*% carbon in 1,000 mg NaCl L^−1^ electrolyte and operating time = 1,200~1,800 s. The standards and low-impact guidelines refer to the MCLs in Ward (2005) and Hickey (2013).

The “steady-state” nitrate-N concentrations in the FCDI system with feed water of different NO_3_-N concentrations are shown in Figure S3. It can be seen that NO_3_-N concentrations in the effluent all decreased with an increase in current density and/or HRT, which is similar to the change of the effluent conductivity (Figure 2). The energy consumption values required to meet different NO_3_-N standards in the three scenarios are provided in Figure 3. For example, at an HRT of 0.98 min, a current input of 20.2 A m^−2^ (i.e., energy consumption = 0.49 kWh m^−3^) is required to reduce the NO_3_-N concentration from 50 to <10 mg L^−1^ that complies with most guideline MCLs (Ward, 2005). However, further decreasing the effluent NO_3_-N concentration to meet higher standards such as 1.0 mg NO_3_-N L^−1^ considered necessary to protect pristine environments with high biodiversity and conservation value (Hickey, 2013) is not without challenges because this would lead to very low effluent conductivity and unnecessary water splitting. In contrast, NO_3_-N concentrations can be reduced to a very low level (<0.1 mg L^−1^) provided that the source water is less contaminated. Specifically, the energy consumptions are 0.90 and 0.46 kWh m^−3^, respectively, for feed waters containing 20 and 10 mg NO_3_-N L^−1^, respectively, at an HRT of 0.98 min (Figure 3). As shown in Figure S3, the “steady-state” effluent concentrations of NO_3_-N all exhibit a current-independent drop, especially evident at low current densities (where electrosorption is less effective) and at longer HRT. A plausible explanation for this effect could relate to the physico-chemical removal of nitrate by the membrane and/or carbon in the system with longer HRT facilitating the mass transfer from feed water to the flow electrode through the anion exchange membrane. As such, consideration was given to the non-electrostatic adsorption of nitrate in FCDI and its impacts on system performance. Unless otherwise stated, the following studies were carried out to treat feed waters initially containing 10 mg NO_3_-N L^−1^ (and 1,000 mg NaCl L^−1^).

### Non-electrostatic Adsorption of Nitrate

**Figure 4:**
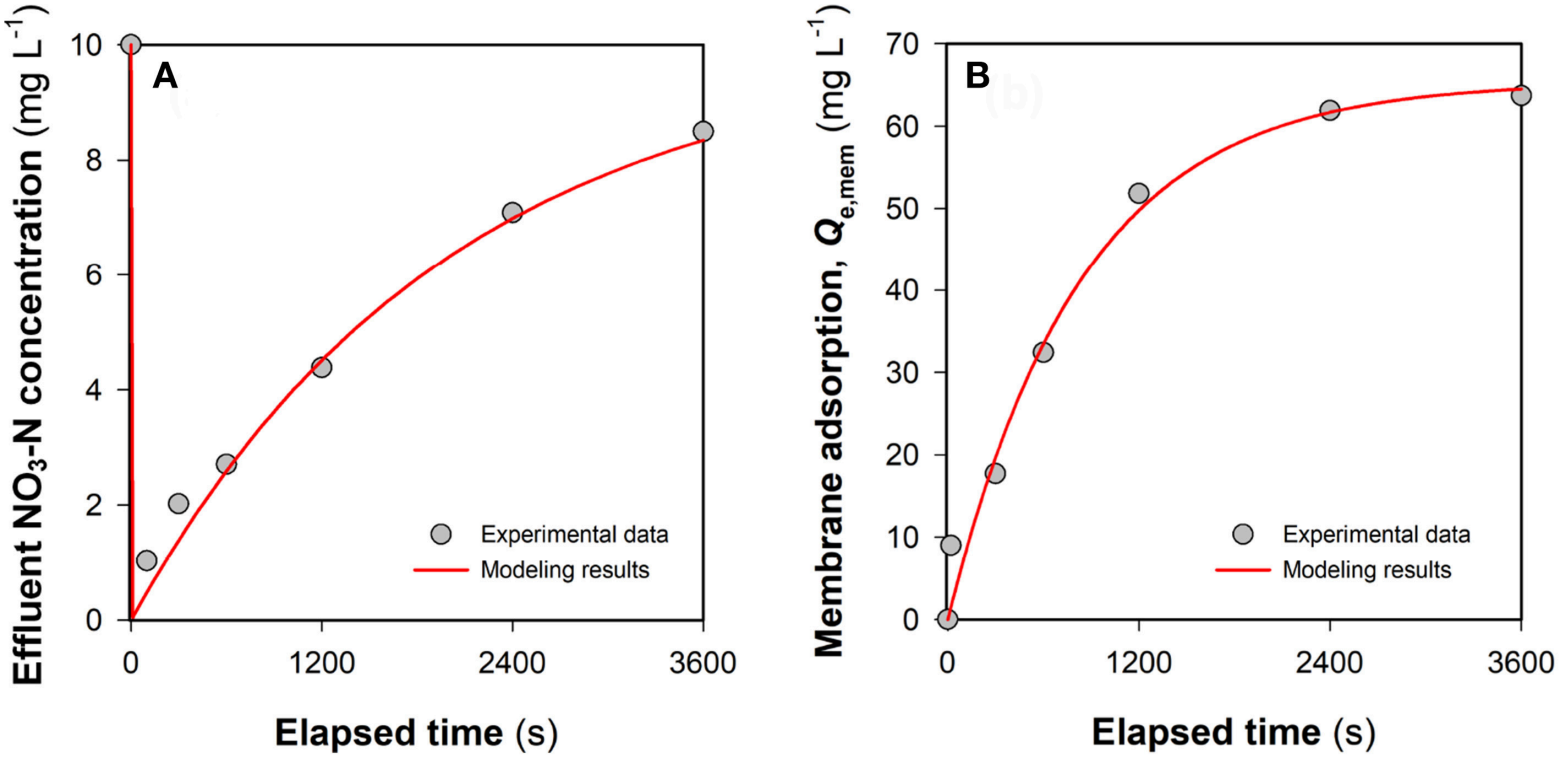
Evaluation of the contribution of membrane adsorption to NO_3_-N removal in FCDI: **(A)** Change of the effluent NO_3_-N concentration and **(B)** mass balance of NO_3_-N removal. The red lines indicate the results of pseudo-first-order kinetic modeling. The system was operated in single-pass mode at *i* = 0 A m^−2^. Experimental conditions: influent NO_3_-N concentration = 10 mg L^−1^ and HRT = 0.98 min.

In an FCDI system, ions in the feed water are expected to migrate through the ion-exchange membranes and end up in the liquid phase of the flow electrode (Ma et al., 2018) or associate with the electrical double layer (EDL) of the carbon electrode particles (Jeon et al., 2013) during electrosorption. While FCDI SCC operation can regenerate the electrode capacitance with most of the EDL-associated ions eventually released into the electrolyte (Yang et al., 2017; He et al., 2018), electrode regeneration might be expected to be compromised under conditions where non-electrostatic adsorption plays a vital role in ion removal. Figure 4A shows the change of the effluent NO_3_-N concentration in the FCDI system in the absence of flow electrode solution and particles and with no applied external electrical field. While rapid decrease in the nitrate concentration was initially observed (as a result, presumably, of nitrate adsorption to the anion exchange membrane), the adsorption sites on the membrane gradually became saturated with the effluent nitrate-N concentration increasing to 8.5 mg L^−1^ after 3,600 s. The physico-chemical adsorption of nitrate onto the membrane can be well described by the pseudo-first-order kinetics model (Equation 4 and Figure 4B). *Q*_*e, mem*_ and *k*_1_ were determined to be 65.4 mg NO_3_-N m^−2^ and 0.0012 s^−1^, respectively. The contribution of membrane adsorption to NO_3_-N removal at time *t* (*X*_IEM_, mg L^−1^) was then estimated (see below) using (Equation 6). Subsequently, a 72 mL saline solution containing 1,000 mg NaCl L^−1^ (i.e., essentially the solution phase of the flow electrode) was added into the electrode chambers. Because of the concentration gradient across the membrane, nitrate in the spacer chamber gradually diffused into the electrolyte circulating in the electrode chambers. As shown in Figure S4A, the effluent NO_3_-N concentration reached the lowest value at 1200 s (7.0 mg L^−1^) and then went up to 8.8 mg L^−1^. There was a good relationship between the total amount of NO_3_-N (i.e., *X*_*tot*_ calculated from Equation 5) removed from the brackish stream and that transferred into the electrolyte (Figure S4B), indicating that (i) the anion exchange membrane was sufficiently saturated following 3,600-s operation (Figure 4A) and (ii) the passive nitrate transfer is negligible when the concentration gradient across the membrane becomes insignificant.

**Figure 5:**
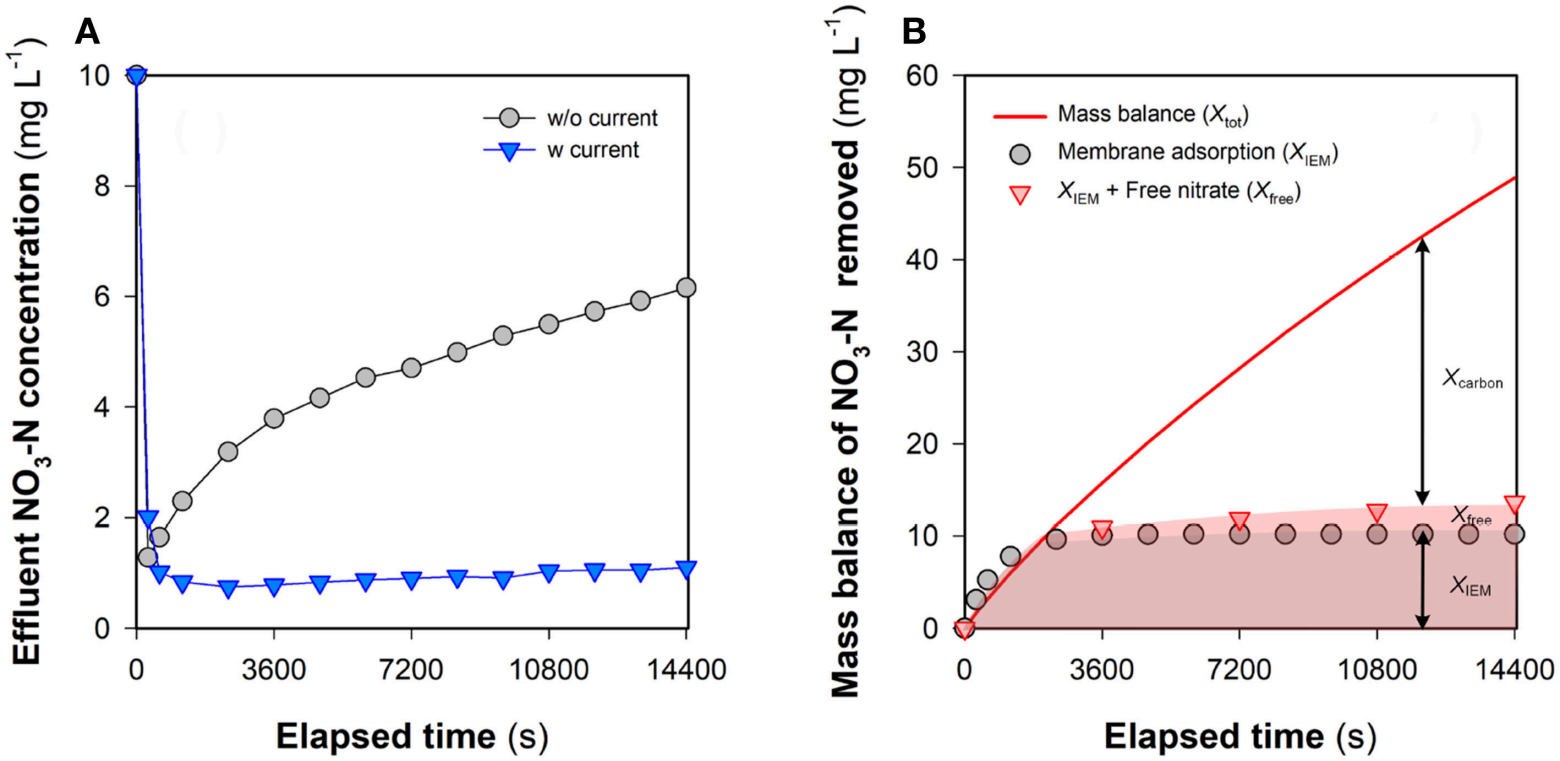
Nitrate removal in FCDI at *i* = 0 A m^−2^. **(A)** Effluent NO_3_-N concentration and **(B)** mass balance of nitrate in the flow electrode. For comparison, the effluent NO_3_-N concentration at 18.6 A m^−2^ is also provided in Figure 5A (blue inverted blue triangles). *X*_*tot*_, *X*_IEM_, *X*_*free*_, and *X*_*carbon*_ were determined according to Equations (5), (6), and (8). Experimental conditions: influent NO_3_-N concentration = 10 mg L^−1^, single-pass, flow electrode = 10 *wt*% carbon in 1,000 mg NaCl L^−1^ electrolyte, and HRT = 0.98 min.

The non-electrostatic contribution of the flow electrode to nitrate removal (*X*_*carbon*_) was evaluated in the FCDI unit by undertaking studies in the absence of an electrical field (*i* = 0 A m^−2^). It can be seen from Figure 5A that the presence of carbon in the flow electrode significantly improves the removal of nitrate, despite the absence of removal via electrosorption. Even though the membrane adsorption capacity was exhausted following 3,600-s operation (Figure 5B), the effluent NO_3_-N concentration was still substantially lower compared to the influent. The theoretical NO_3_-N concentration in the flow electrode (*X*_*tot*_) and the concentration of nitrate on the membrane (*X*_*IEM*_) were, respectively, calculated according to Equations (5) and (6) with *X*_*free*_ determined based on the aqueous NO_3_-N concentration in the flow electrode at time *t*. Results in Figure 5B suggest that a large fraction of NO_3_-N removed in FCDI should not be ascribed to *X*_*IEM*_ and *X*_*free*_, with this result highlighting the importance of non-electrostatic adsorption of nitrate to the carbon particles present in the flow electrode chamber. As such, the adsorption isotherm of nitrate from aqueous solutions by activated carbon was measured at room temperature (Figure S5), with the experimental data fitting well with the Langmuir model (Equation 7). Parameters *Q*_∞_ (1.10 mg g^−1^) and *b* (0.073 L mg^−1^) were then used to characterize the nitrate removal by carbon adsorption (*X*_*carbon*_) in FCDI (Equation 8). Mechanisms that may contribute to the non-electrostatic adsorption process include exchange between nitrate and (i) ions associated with polar functional groups on the carbon surface and/or (ii) ions in the EDLs of the carbon in the flow electrode (Gierak and Lazarska, 2017). Both mechanisms could negatively influence the effectiveness of FCDI for nitrate removal; for example, the exhaustion of active sites on the carbon particle surface may result in deterioration of process performance, and the pre-accumulation of nitrate in the EDLs would be expected to affect the capacitive kinetics of nitrate removal when an electrical field is applied.

### Comparison of *X*_IEM_, *X_*Free*_,* and *X_*Carbon*_* During Nitrate Removal

**Figure 6:**
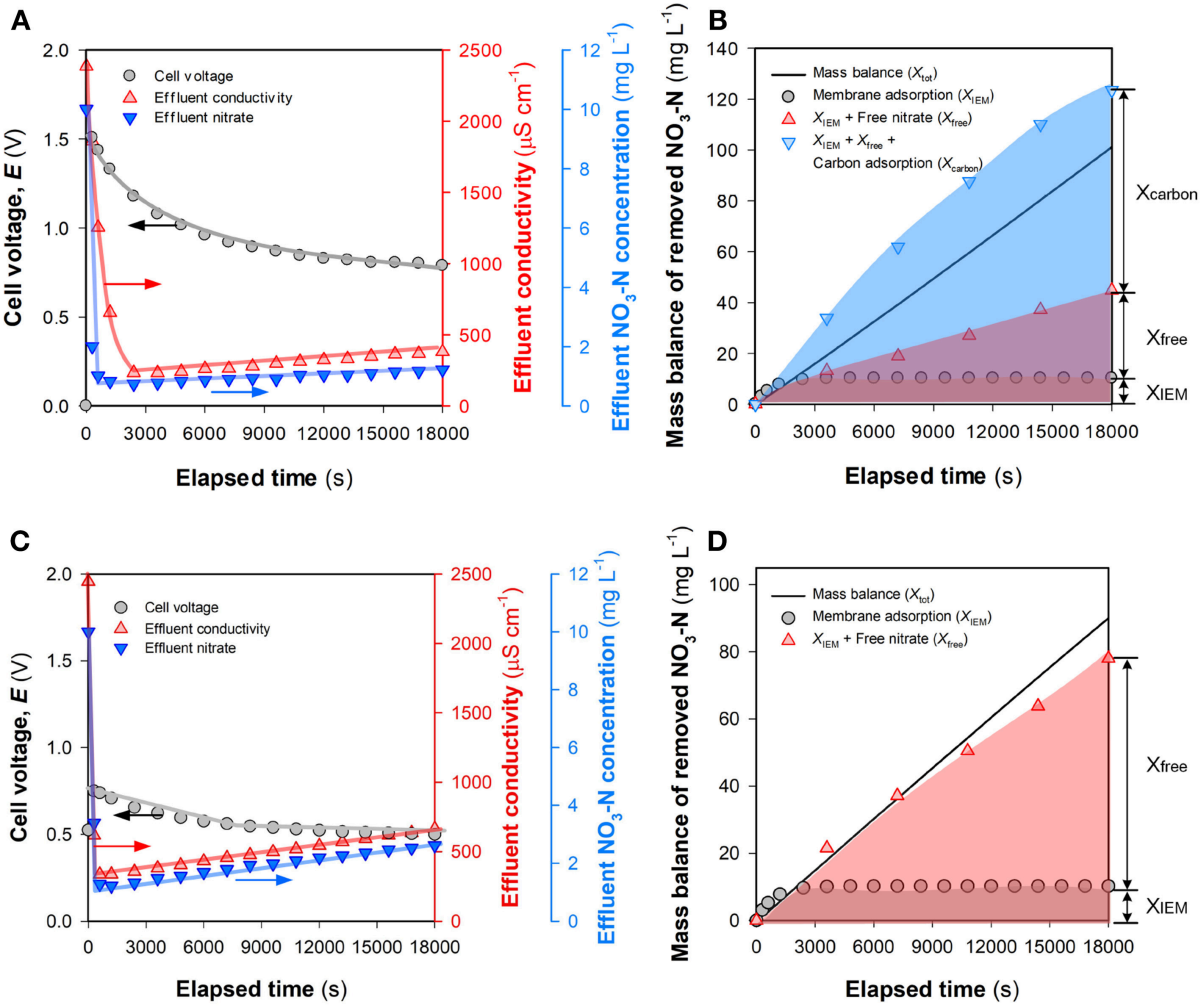
Comparison of *X*_IEM_, *X*_*free*_, and *X*_*carbon*_ in nitrate removal in FCDI. **(A)** Change of voltage and treatment efficiency and **(B)** mass balance of nitrate removed with the electrolyte in flow electrode consisting of 1,000 mg NaCl L^−1^. **(C)** Change of voltage and treatment efficiency and **(D)** mass balance of nitrate removed with the electrolyte in flow electrode consisting of 100 mg NO_3_-N L^−1^ and 1,000 mg NaCl L^−1^. Experimental conditions: influent NO_3_-N concentration = 10 mg L^−1^, single-pass, HRT = 0.98 min, and *i* = 18.6 A m^−2^.

Figure 6A depicts the time-course results of salt and nitrate removal in the FCDI system under constant current mode. The effluent conductivity and NO_3_-N concentration initially decreased to very low levels. Subsequently, slight deterioration in ion removal efficiency was observed with the effluent NO_3_-N concentration gradually exceeding the MCL (1 mg L^−1^) for environments with high biodiversity and conservation value (Hickey, 2013). An overview of the fate of nitrate within the FCDI system is presented in Figure 6B. The theoretical NO_3_-N removal amount (*X*_IEM_ + *X*_*free*_ + *X*_*carbon*_) is estimated to be a little higher than the experimentally determined value (*X*_*tot*_) with this discrepancy likely ascribed to the fact that *X*_*carbon*_ was estimated under assumed equilibrium conditions (Equation 8). In SCC FCDI, the flow electrodes are continuously charged in the cell and re-generated in the shared reservoir (Figure 1) with ions in the electrical double layers (EDLs) released into the electrolyte when discharging occurs in the shared reservoir (Yang et al., 2017; He et al., 2018). The ions released during this discharging process may be re-immobilized on the carbon particles once they re-enter to charged environment of the electrode flow channels. As such, aqueous NO_3_-N concentration in the flow electrode is constantly changing with the short contact time between carbon and electrolyte in the shared reservoir likely insufficient for the physico-chemical adsorption process to achieve equilibrium with the result that the calculated *X*_*carbon*_ was, in all likelihood, somewhat higher than the actual amount of nitrate adsorbed on the carbon. Overall, *X*_IEM_ became insignificant at extended times (Figure 6B) although it is expected that the membrane's affinity for nitrate will continue to facilitate the selective transfer of nitrate across the membrane. *X*_*carbon*_ accounted for a major part of the nitrate removed with results in Figure S6 indicating that nitrate on the carbon is difficult to be electrostatically desorbed, even when the current is reversed. Therefore, consideration was given to the question of how the FCDI performance changes once the capacity of the carbon electrode particles to non-electrostatically adsorb nitrate has been exhausted.

A flow electrode suspension consisting of 10 *wt*% carbon materials, 100 mg NO_3_-N L^−1^ and 1,000 mg NaCl L^−1^ was continually mixed on a magnetic stirrer for at least 12 h prior to use in order to achieve adsorption equilibrium. Figures 6C,D summarize the temporal change of the treatment efficiency and mass balance of nitrate on using this “nitrate-equilibrated” suspension in the flow electrode. Compared to the results in Figure 6A, while a similar trend in the variations of effluent conductivity and NO_3_-N concentration was observed, the FCDI efficiency decreased due to the presence of nitrate in the flow electrode. For example, the lowest effluent NO_3_-N concentration in Figure 6C was 1.22 mg L^−1^ at 1,200 s, consistently higher than that in Figure 6A. In this latter case, the sum of *X*_IEM_ + *X*_*free*_ provide a reasonable explanation for *X*_*tot*_, indicating that the formation of EDLs in SCC FCDI was less affected due to the exhaustion of the physico-chemical adsorption capacity of the carbon particles in the flow electrode chamber. While one might speculate that nitrate migration under this condition is essentially an electrodialysis process, previous studies have confirmed that the capacitive mechanism dominates in SCC FCDI (i.e., *X*_*free*_) in view of the much lower efficiency observed when only electrodialysis occurs (He et al., 2018). Nevertheless, the effluent NO_3_-N concentration exceeded 1 mg L^−1^ over the whole experiment (Figure 6C) and, even when *X*_*carbon*_ contributed to nitrate removal, deterioration in the process performance still occurred (Figure 6A). It should be noted that as a result of nitrate (and salt) transfer into the electrolyte, the ionic resistance in the flow electrode constantly decreased during the desalting process. The cell voltage in Figure 6C (0.75~0.50 V) is much lower than that in Figure 6A (1.5~0.79 V). In classical CDI (and MCDI), a constant effluent salt concentration could be achieved under constant current mode, largely due to the buildup of electrical potential at the Stern plane (Δϕ_*d*_) (Tsouris et al., 2011; Zhao et al., 2012; Tang et al., 2016). According to the modified Donnan model (Porada et al., 2013; Wang et al., 2018), there is a positive correlation between the excess salt adsorption (Γ) and both the effective micropore volume (relative to total electrode volume) (*p*_*mi*_) and the non-dimensional Donnan potential within the micropores (Δϕ_*D*_ that relates to Δϕ_*d*_). With regard to charge neutralization and electrode re-generation in SCC FCDI, constant *p*_*mi*_ would be expected to be maintained during the desalting process. However, the decrease in cell voltage when the FCDI system is operated in constant current mode would lead to the re-distribution of electrical potential with subsequent shrinkage in ϕ_*d*_. Therefore, stable effluent quality cannot be guaranteed for SCC FCDI when operated in constant current mode.

### Critical Parameters Influencing FCDI Performance Under Continuous Operation

To obtain a stable desalting performance, the FCDI system was operated in constant voltage mode with the charging voltage (1.0 V) comparable to the initial cell voltage under the constant current mode at *i* = 18.6 A m^−2^ (Figure 6A). It can be seen from Figure S7 that, with the migration of ions into the flow electrode, the current in the circuit gradually increased due to the decrease in the internal ionic resistance (Dykstra et al., 2016). A fairly stable effluent quality (NO_3_-N < 1 mg L^−1^) was obtained when operated in constant voltage mode, with the results in reasonable agreement with those in the initial stage of operation under constant current mode (Figure S3). As such, operation in constant voltage mode is recommended for SCC FCDI in water purification.

**Figure 7:**
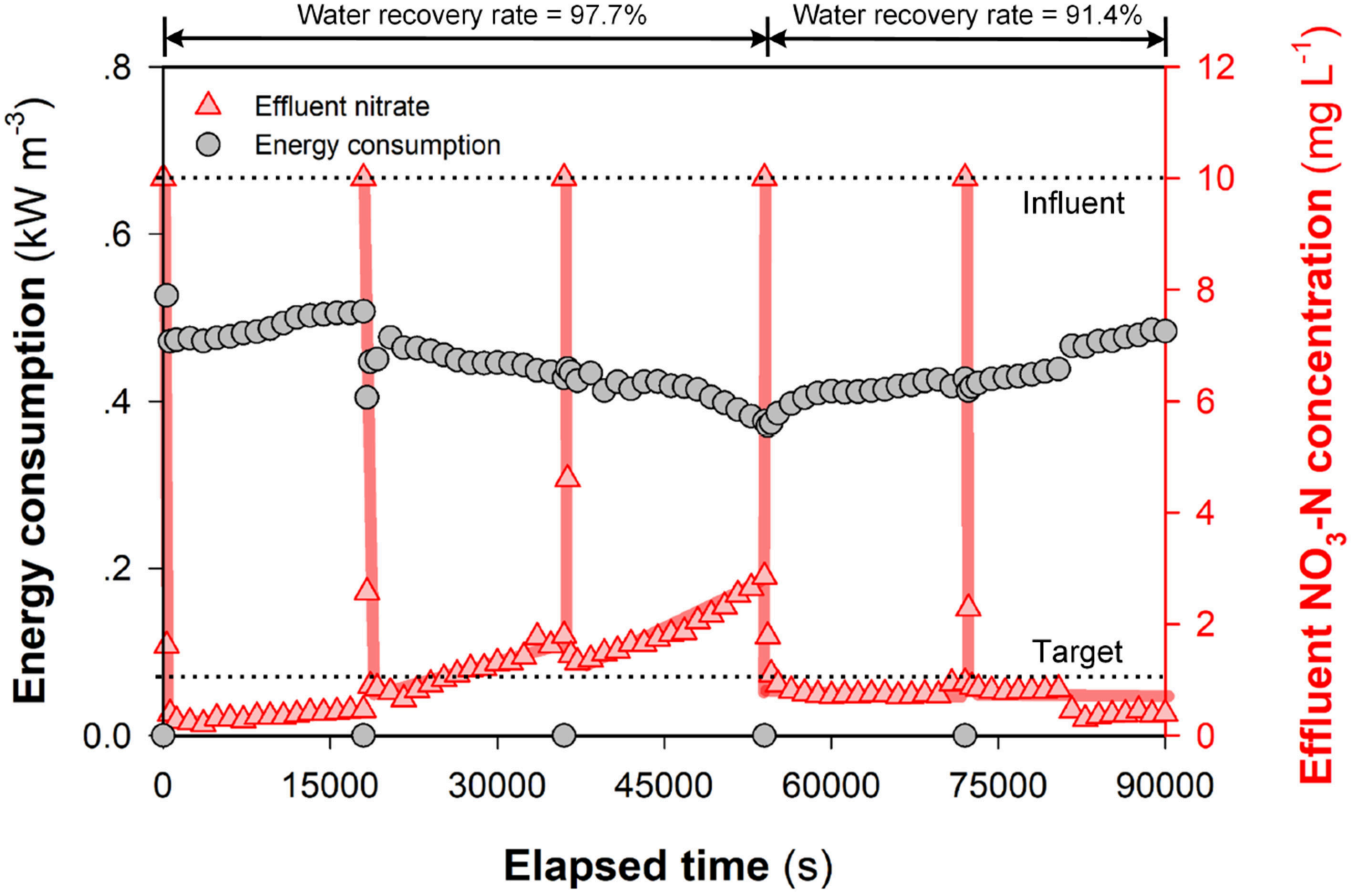
Energy consumption and nitrate removal efficiency of SCC FCDI at different water recovery rates. Experiments were conducted following that in Figure S7 to reduce *X*_*carbon*_. The electrolyte was partially replaced with 1,000 mg NaCl L^−1^ solution every 5 h. Experimental conditions: initial NO_3_-N concentration = 10 mg L^−1^, single-pass, cell voltage = 1.0 V and HRT = 0.98 min. Lines serve to guide the eye.

Specific energy consumption and water recovery are two key indicators that may be used to evaluate the performance of (M)CDI with regard to removal of ions from contaminated water sources (Suss et al., 2015). While one advantage of FCDI is the continuous desalting operation, the generation of a waste stream in SCC FCDI relates to the accumulation of ions in the liquid phase of the flow electrode (Doornbusch et al., 2016) that likely results in (i) water transfer into and dilution of the carbon content in the flow electrode (Yang et al., 2017) and (ii) back-diffusion and/or leakage of ions. Dilution of the carbon content will result in a decrease in the charge transfer efficiency within the flow electrodes and leakage of co-ions inevitably leads to a reduction in of average salt removal rate and decrease in Coulombic efficiency (Tedesco et al., 2016). Using the definition of SCC FCDI water recovery rate (γ) provided in Equations (9) and (10), we compare the performance (with regard to both effluent nitrate concentration and energy consumption) for water recovery rates of 97.7 and 91.4% (see Figure 7 and Figure S8). At the extremely high water recovery rate (97.7%), the current density initially increases with ion migration into the solution phase of the flow electrode (Figure S8). However, the over accumulation of ions in the flow electrode likely results in an increase in the internal (polarization) resistance with the subsequent current density and nitrate removal efficiency decreasing over time. It is also possible that there is an excess number of ions in the electrolyte compared to the available sites on the carbon (*p*_*mi*_) at extremely high water recovery rates with this leading to the less-effective electrodialysis process playing a more important role in ongoing ion removal. In comparison, SCC FCDI works well at a “relatively lower” water recovery rate of 91.4% (still significantly higher than (M)CDI) (Porada et al., 2013; Suss et al., 2015). The effluent NO_3_-N concentration was constantly lower than 1 mg L^−1^ at reasonable energy consumption (~0.5 kWh m^−3^) and productivity (HRT < 1 min) (Figure 7).

## Conclusions

In this study, SCC FCDI was used to remove nitrate from source waters containing different nitrate concentrations. Results indicate that FCDI is well suited to removing nitrate to levels consistent with extremely stringent standards (<1 mg NO_3_-N L^−1^) though a less onerous target of, say, 10 mg L^−1^ would be more cost-effective (i.e., ~0.4 kWh m^−3^) under conditions where the influent nitrate concentration is high (50 mg NO_3_-N L^−1^). Investigation of the fate of nitrate indicated that non-electrostatic adsorption of nitrate to the carbon particles initially plays a vital role in nitrate removal in FCDI. Nevertheless, the exhaustion of non-electrostatic adsorption capacity with ongoing operation did not lead to significant deterioration in performance with this result likely ascribed to the continued retention of nitrate in the EDLs of the carbon particles in the flow anode. In continuous operation of SCC FCDI, constant voltage mode is better suited to maintaining stable effluent quality. Through periodic replacement of the electrolyte at a water recovery of 91.4%, the FCDI system can achieve a continuous desalting performance with the effluent NO_3_-N concentration below typical target MCLs at low energy consumption (~0.5 kWh m^−3^) but high productivity (HRT < 1 min).

## Author Contributions

All authors contributed to the planning of this work while JS, JM, and CZ undertook the experimental investigations. All authors contribute to preparation and editing of the manuscript.

### Conflict of Interest Statement

The authors declare that the research was conducted in the absence of any commercial or financial relationships that could be construed as a potential conflict of interest.
