# Supplementary material for: Implication of Non-electrostatic Contribution to Deionization in Flow-Electrode CDI: Case Study of Nitrate Removal From Contaminated Source Waters
Source: Front Chem. 2019 Mar 22;7:146. doi: 10.3389/fchem.2019.00146 (PMC6439345; doi:10.3389/fchem.2019.00146)
Supplement: Supplementary file 1 [file Table_1.DOCX]

**Supporting Information**

**Implications of Non-electrostatic Adsorption to Deionization in Flow-electrode CDI: Case Study of Nitrate Removal from Brackish Source Waters**

Jingke Song^1,2,3†^, Jinxing Ma^1†^, Changyong Zhang^1^, Calvin He^1^, and T. David Waite^1*^

*^1^UNSW Water Research Centre, School of Civil and Environmental Engineering, University of New South Wales, Sydney, NSW 2052, Australia*

*^2^College of Environmental Science and Engineering, Tongji University, Shanghai* *200092, P.R. China*

*^3^Shanghai Institute of Pollution Control and Ecological Security, Shanghai 200092, P. R. China*

Email addresses: [jingke.song@unsw.edu.au](mailto:jingke.song@unsw.edu.au) (Jingke Song); [jinxing.ma@unsw.edu.au](mailto:jinxing.ma@unsw.edu.au) (Jinxing Ma); [changyong.zhang@unsw.edu.au](mailto:changyong.zhang@unsw.edu.au) (Changyong Zhang); [calvin.he1@unsw.edu.au](mailto:calvin.he1@%20unsw.edu.au) (Calvin He); [d.waite@unsw.edu.au](mailto:d.waite@unsw.edu.au) (T. David Waite)

† These authors contributed equally to this work.

***Corresponding author:** E-mail [d.waite@unsw.edu.au](mailto:d.waite@unsw.edu.au); Tel. +61-2-9385-5060


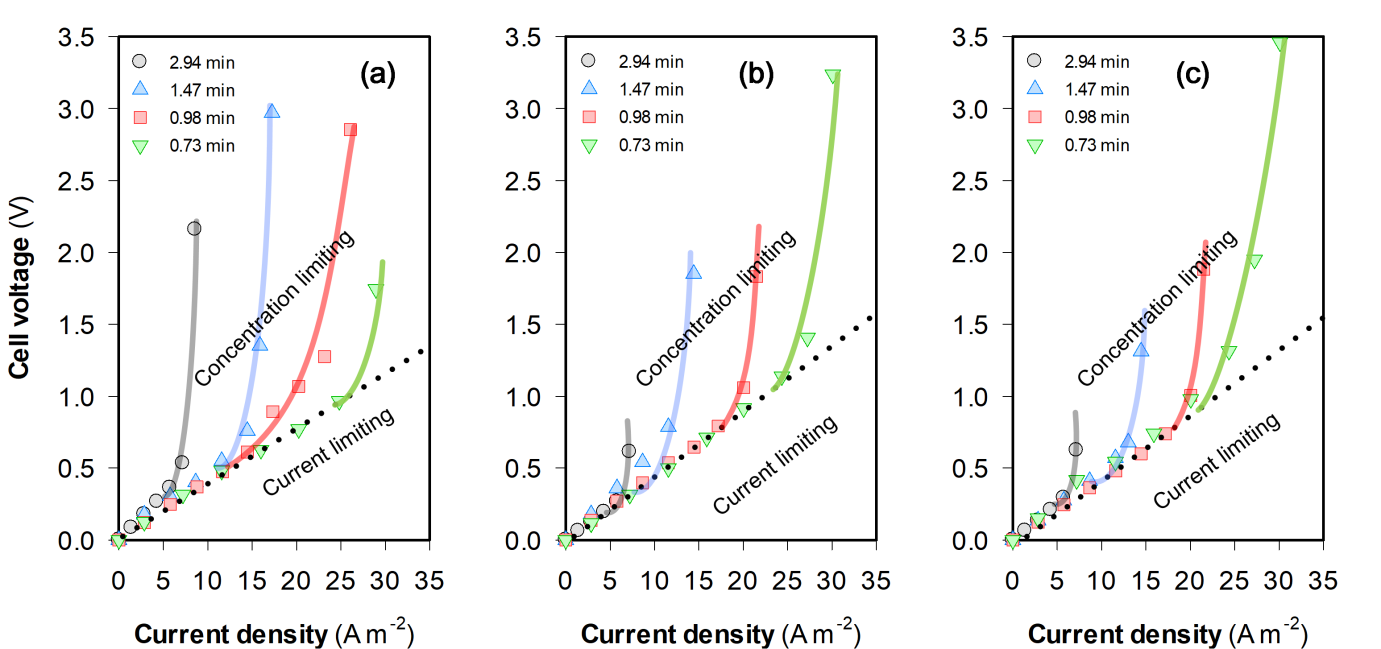


**Fig. S1.** Change of the average cell voltage as a function of the current density. Initial influent NO_3_-N concentration: (a) 50, (b) 20 and (c) 10 mg L^−1^. The legends in figures indicate different HRTs. Experimental conditions: single-pass, constant-current mode and operating time = 1200 ~ 1800 s. Lines serve to guide the eye.

**
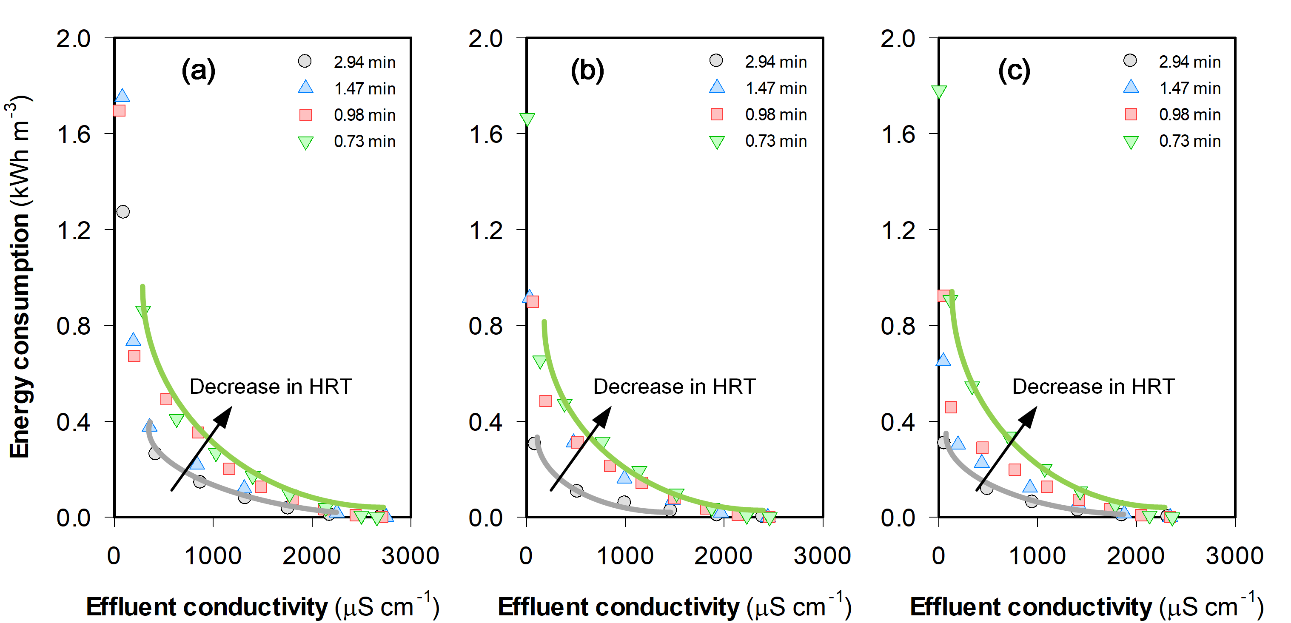
**

**Fig. S2.** Energy consumption to achieve different effluent conductivity at an influent NO_3_-N concentration of (a) 50, (b) 20 and (c) 10 mg L^−1^. The legends in figures indicate different HRTs. Experimental conditions: single-pass, constant-current mode and operating time = 1200 ~ 1800 s. Lines serve to guide the eye.


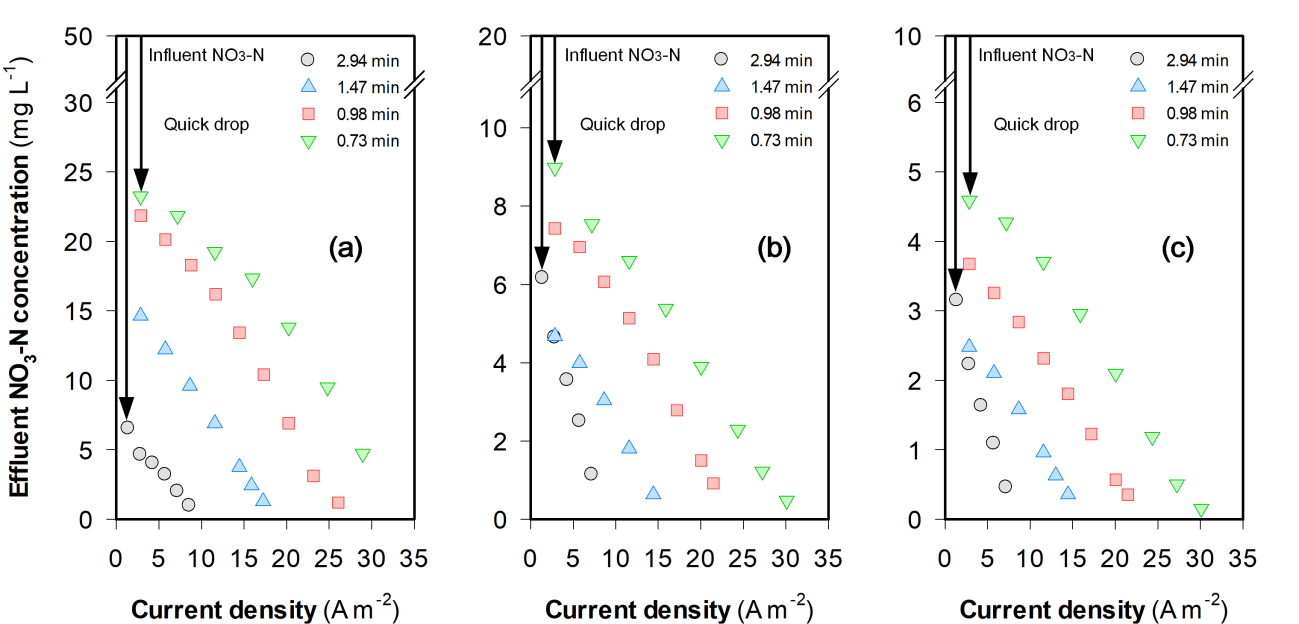


**Fig. S3.** Change of the effluent NO_3_-N concentration at an influent NO_3_-N concentration of (a) 50, (b) 20 and (c) 10 mg NO_3_-N L^−1^. All experiments were conducted in single-pass, constant-current mode. The legends in figures indicate different HRTs. The operating time for electrosorption is 1200 ~ 1800 s.

**
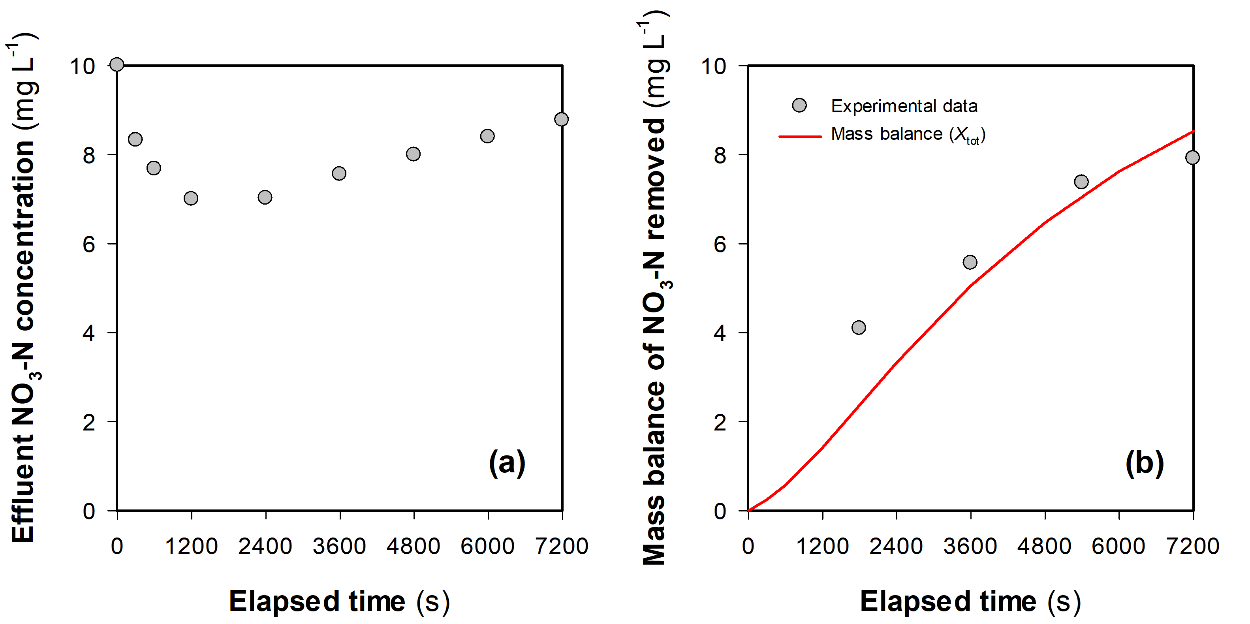
**

**Fig.** **S4.** Diffusion of nitrate into the solution phase of the flow electrode as a result of the concentration gradient. (a) The time-course results of the effluent NO_3_-N concentration and (b) mass balance of NO_3_-N removal in the flow electrode initially containing 1000 mg NaCl L^−1^. The experiment was carried out following the membrane reaching the adsorption capacity (as shown in Fig. 4). The red line in SI Fig. S4b stands for the results according to the mass balance calculation (Eq. 5). Experimental conditions: influent NO_3_-N concentration = 10 mg L^-1^, *i* = 0 A m^−2^, single-pass and HRT = 0.98 min.

**
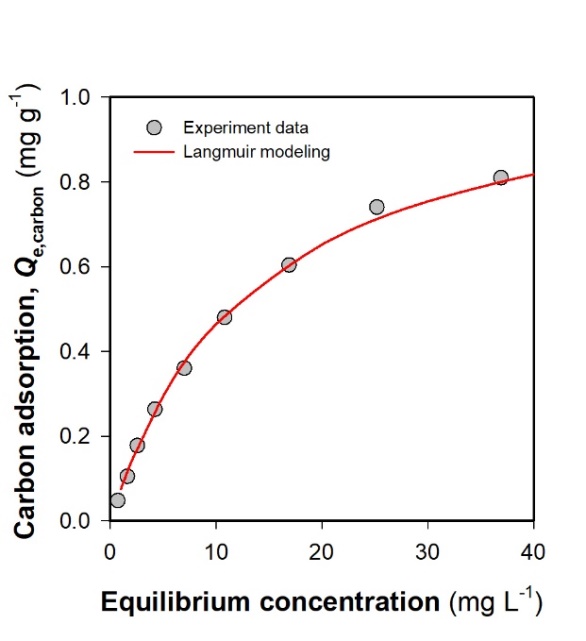
**

**Fig. S5.** Langmuir modeling of nitrate adsorption by the active carbon materials in the flow electrode at room temperature.


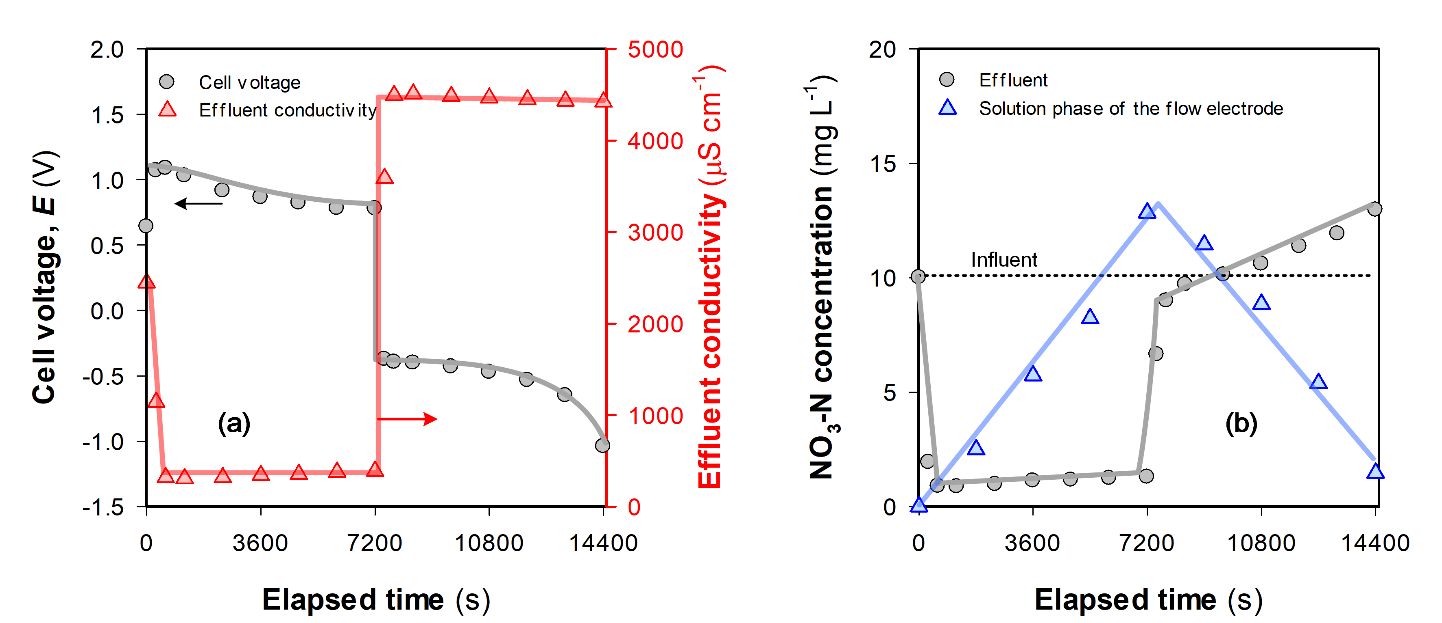


**Fig. S6.** Electro-desorption of NO_3_-N in the carbon by reversing the current. (a) Change of cell voltage and effluent conductivity and (b) change of the concentrations of effluent nitrate and nitrate in the liquid phase of flow electrode. The effluent NO_3_-N concentration was only slightly higher than the influent, indicating that nitrate on the carbon was difficult to be electrostatically desorbed. Experimental conditions: initial NO_3_-N concentration = 10 mg L^−1^, single-pass, HRT = 0.98 min, *i* = 18.6 A m^−2^ from 0 to 7200 s and -18.6 A m^−2^ from 7200 to 14400 s.

**
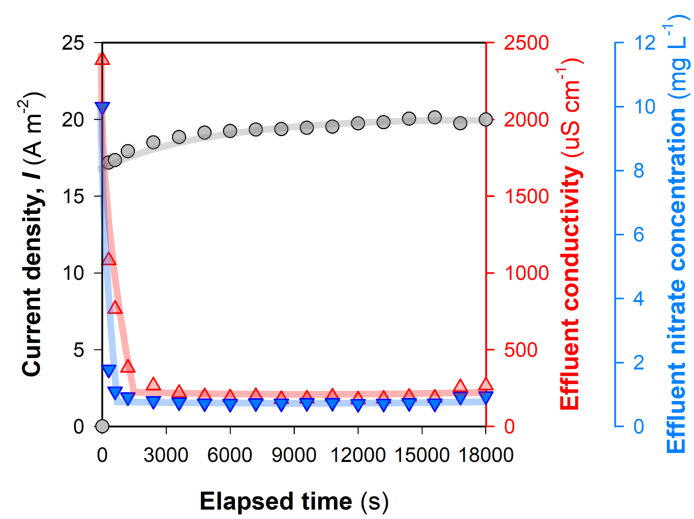
**

**Fig. S7.** Operation of FCDI in constant voltage mode. Experimental conditions: influent NO_3_-N concentration = 10 mg L^−1^, single-pass, HRT = 0.98 min and cell voltage = 1.0 V.


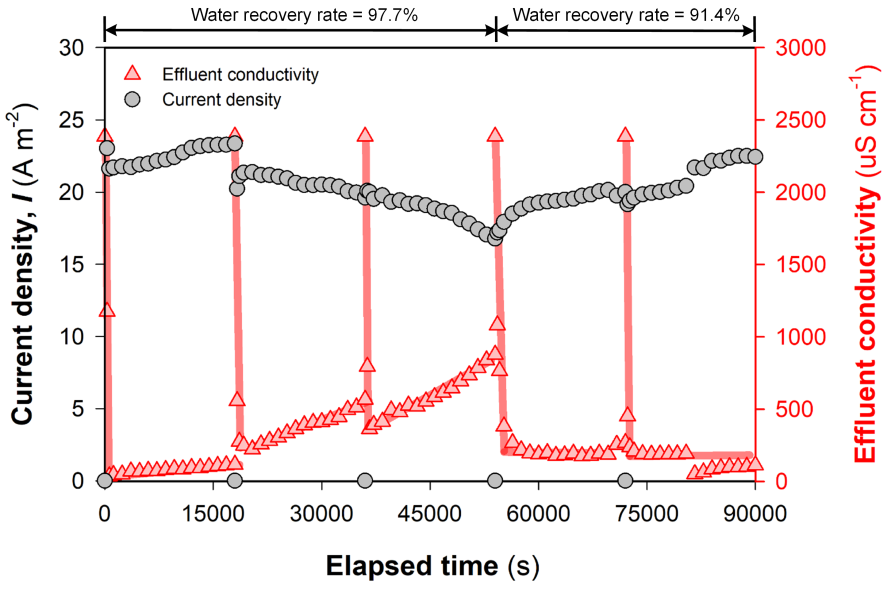


**Fig. S8.** Current density and effluent conductivity of SCC FCDI at different water recovery rates. Experiments were conducted following that in SI Fig. S7 to reduce *X_carbon_*. The electrolyte was partially replaced with 1000 mg NaCl L^−1^ solution every 5 hours. Experimental conditions: initial NO_3_-N concentration = 10 mg L^−1^, single-pass, cell voltage = 1.0 V and HRT = 0.98 min. Lines serve to guide the eye.
